# Selective Laser Melting of 18NI-300 Maraging Steel

**DOI:** 10.3390/ma13194268

**Published:** 2020-09-25

**Authors:** Mariusz Król, Przemysław Snopiński, Jiří Hajnyš, Marek Pagáč, Dariusz Łukowiec

**Affiliations:** 1 Department of Engineering Materials and Biomaterials, Faculty of Mechanical Engineering, Silesian, University of Technology, 44-100 Gliwice, Poland; przemyslaw.snopinski@polsl.pl (P.S.); dariusz.lukowiec@polsl.pl (D.Ł.); 2 Center of 3D Printing Protolab, Department of Machining, Assembly and Engineering Technology, Faculty of Mechanical Engineering, VSB-TU Ostrava, 17. Listopadu 2172/15, 708-00 Ostrava-Poruba, Czech Republic; jiri.hajnys@vsb.cz (J.H.); marek.pagac@vsb.cz (M.P.)

**Keywords:** 18Ni-300 maraging steel, SLM, 3D printing parameters, relative density, structure

## Abstract

In the present study, 18% Ni 300 maraging steel powder was processed using a selective laser melting (SLM) technique to study porosity variations, microstructure, and hardness using various process conditions, while maintaining a constant level of energy density. Nowadays, there is wide range of utilization of metal technologies and its products can obtain high relative density. A dilatometry study revealed that, through heating cycles, two solid-state effects took place, i.e., precipitation of intermetallic compounds and the reversion of martensite to austenite. During the cooling process, one reaction took place (i.e., martensitic transformation), which was confirmed by microstructure observation. The improvements in the Rockwell hardness of the analyzed material from 42 ± 2 to 52 ± 0.5 HRC was improved as a result of aging treatment at 480 °C for 5 h. The results revealed that the relative density increased using laser speed (340 mm/s), layer thickness (30 µm), and hatch distance (120 µm). Relative density was found approximately 99.3%. Knowledge about the influence of individual parameters in the SLM process on porosity will enable potential manufacturers to produce high quality components with desired properties.

## 1. Introduction

Since 1950, maraging steel has been intensively subjected to research and development. It belongs to the group of high strength materials with broad application in many different industries. The mechanical properties of maraging steel can be formed due to proper heat treatment. The increased strength of maraging steel is obtained via intermetallic phase precipitation, resulting in improvements of hardness, ductility, and strength after suitable heat treatment. The main alloy components of maraging steel are nickel, cobalt, and molybdenum, as well as a low content of carbon [1,2]. The 18Ni-300 steel is generally heat treated, hardened and then aged in a temperature range of 450–510 °C, which results in improved mechanical properties (i.e., hardness and strength as nucleation effect [3,4] like *γ*-Ni_3_Mo, NiAl, Ni_3_(Al, Ti), Ni_3_(Ti, Mo), Ni(Al, Fe), *η*-Ni_3_Ti, Fe_2_Mo, *σ*-FeMo, *µ*-Fe_7_Mo_6_, FeTi, Fe_2_Ti, and Ni_3_Al [5,6]). As a result of such research, the development [7,8,9] and optimization of chemical compositions and heat treatment processes has distinguished groups of maraging steel 18Ni-300, due to particularly high strength properties that exceed 2000 MPa of tensile strength.

Among machine part manufacturing technology, there is a common interest in additive manufacturing technology, which includes selective laser melting (SLM) [10,11,12,13,14]. In this method, a focused laser beam is used to spot powder beds and perform successive melting of the powder layer that bonds to current layers, which results in a 3D model. The SLM technique has many benefits over other parts manufacturing technologies, including considerable material utilization, easy and healthy product design, good part and production flexibility, and functional features of the obtained components that can be applied to the production parameters [4,15,16]. Correct selection of SLM process parameters allows one to obtain the desired structure and surface quality of the manufactured element. The conditions of the additive production process are determined by the heat transfer process parameters due to powder beds and metal powder physical attributes. Process parameters include laser power, focused spot diameter, exposure time, point distance, hitch distance, layer thickness, scan speed, base plate temperature, and protective atmosphere. Material properties also have a significant influence on obtaining properties, including grain diameter, thermal conductivity, thermal diffusivity, and specific heat capacity. The desirable properties of the finished product after the SLM process is the apparent density of the model as close as possible to the actual material density, without closed and open porosity. This is possibly due to the smooth external surface of the model, no deformation of the model’s geometry, small grain size (<1 µm), high hardness, and tensile strength obtained directly after the SLM process. One of the most important parameters that determines model quality is energy density, which is dependent on process parameters [4,12,16]. The success of 3D models depends on adjusting parameters to the energy demand needed, especially concerning the phenomenon of melting powder bed layers and obtaining high material density [17,18].

The energy density (*E_d_*) proposed by Casolino et al. [17] and Campanelli et al. [19] is determined by Equation (1):

(1) $$E_{d}=\frac{P}{V\cdot d}\;\left(\frac{J}{{\mathrm{mm}}^{2}}\right)$$

where a relation between laser power (*P*), scan speed (*V*), and spot diameter (*d*) is shown. In this approach, the highest density, low roughness, and good hardness of the material can be obtained with an energy density higher than 2 J/mm^2^. Moreover, Kempken et al. [20] confirmed that increasing the scan speed (*V*) reduces both hardness and density of printing material. Bhardwaj et al. [5] described energy density using Equation (2):

(2) $$E_{d}=\frac{P}{V\cdot h\cdot t}\;\left(\frac{J}{{\mathrm{mm}}^{3}}\right)$$

where energy density is proportional to laser power (*P*) and inversely proportional to scan speed (*V*), hatch distance (*h*), and layer thickness (*t*). Yuchao et al. [21] calculated the energy density by considering laser power (*P*), spot radius (*r*), scan speed (*V*), and scanning space (*s*) in Equation (3):

(3) $$\psi =\frac{P}{\pi r^{2}}\cdot \frac{2r}{v}\cdot \frac{2r}{s}=\frac{4P}{\pi vs}$$

Increasing the laser power should linearly increase the amount of energy delivered to the sample’s surface, as well as the exposure time of the spot laser beam on powder beds. However, increasing the scanning speed, spot diameter, hitch distance, and point distance should decrease energy density, which may cause a lower density of the material. Lower scan speeds allow one to obtain better relative density. Bei et al. [21] showed that too low scanning speed had a negative effect on the solidification of liquid metal, especially in overlapping arrangements of the laser spot. The reason for this is liquid metal rapidly reheating, which leads to spattering and void formation.

Considering the above factors that affect the properties of the obtained component, it is also necessary to find the components’ producing time. The primary variables that optimize time when manufacturing parts are hatch distance ∆*y_s_*, layer thickness *D_s_*, and scanning velocity *V_scan_*. The thickness of the deposited layer and scanning speed is limited mainly by laser parameters. The beam diameter defines the hatch distance and typically equals approximately 0.7 times the beam diameter [22]. From Equation (4), the efficiency of the SLM process can be quantified.

(4) $$V=D_{s}\cdot ∆y_{s}\cdot V_{scan}$$

In the present work, steel 18Ni-300 was processed using the SLM technique to determine changes in densification behavior, microstructure, and hardness, using various process conditions, while maintaining a constant level of linear energy density.

## 2. Materials and Methods

In this work, we analyzed and processed a 18Ni-300 maraging alloy (1.2709) supplied by BÖHLER (Kapfenberg, Austria) as a gas-atomized powder, with a grain size in the range of 15–45 μm and spherical form. The chemical composition delivered and used in the experiments is shown in Table 1. It was found that the chemical compositions of as-fabricated specimens were almost the same as the declared requirements. Moreover, there were no visible elemental content distinctions within the powder and as-fabricated components, which designated that the burning loss and alloying elements evaporation during laser irradiation were negligible.

The particle size distribution was determined using laser diffraction via the Frauenhofer model for particle light scattering. Our analysis was performed using wet dispersion via Analysette 22 MicroTec apparatus, Fritsch GmbH (Sonneberg, Germany).

The selected technological properties of powder Ni-18 M300 steel are flowability, bulk density, and tapped density. Each was measured using a Hall Flowmeter funnel according to ISO 3923-2 standard [23].

The morphology of delivered steel powder and microstructure of fabricated samples was analyzed on a scanning electron microscope (SEM) Supra 35 of Zeiss Company (Oberkochen, Germany). To capture the structures of the analyzed components, a secondary electron detection (SE) was implemented, with an accelerating voltage of 20 kV.

For metallographic examination, a light optical microscope (LOM) was used on the as-built and heat treated components. Metallographic specimens were fabricated using a conventional metallographic specimen preparation method that consisted of grinding, emery paper polishing, and cloth polishing. The LOM-based observations were made at the metallographic specimens etched with 5% Nital solution (95 mL HCl + 5 mL HNO_3_). The light optical microscopy was carried out using a Leica Image Analyzer (Vienna, Austria) connected to a personal computer with image analysis software. The density of the manufactured components was estimated using an image analysis method to assesses the porosity within a sample by measuring the percentage area of porosity on the polished surfaces. The average values and standard deviations were estimated based on the observation of five different regions.

The AM125 system from RENISHAW (New Mills, UK) was utilized to manufacture components via the SLM technique. The system is characterized by Ytterbium (Yb) fiber laser of maximum laser power (200 W), scan speed (2000 mm/s), and wavelength (1074 nm). The designed components were manufactured on a mild steel platform at an oxygen level below of 500 ppm. The preheating of the substrate was not applied. Samples were manufactured using a meander scanning strategy that changed the scan direction angle of each subsequent layer by 67°. Many recent researches have been carried out to examine the influence of manufacturing parameters with the SLM technique for materials made of M300 steel. However, not a single work has presented the effect of manufacturing parameters while maintaining a constant energy density. Different producers of SLM machines recommend different process parameters to achieve full density components. For example, for M300 steel, EOS recommends applying printing parameters at a layer thickness of 30 μm, whereas TRUMPF recommends 50 μm of layer thickness. Renishaw did not develop printing parameters for the AM125 machine. Thus, the central idea of the present study was to present an influence of process parameters for the SLM technique on relative porosity and hardness of components manufactured via the SLM technique, while maintaining constant energy density at approximately 160 J/mm^3^, which is what was recommended by BÖHLER, the producer of the powder. The energy density (*E*) is a critical parameter in the SLM technique. It correlates to laser power (*P*), scan speed (*V*), hatch distance (*h*), and layer thickness (*t*) and, herein, was determined using Equation (2).

Table 2 lists different process conditions, which offer the possibility to generate constant energy density (approximately 160 J/mm^3^). The powder beds were selectively fused layer by layer until the final 3D component was finished. The rest of the process conditions used in this experiment were constant, i.e., laser power 200 W, a laser spot diameter of 35 µm, exposure time of 100 μs, and point distance of 60 μm.

Dilatometric measurements, i.e., heating and cooling cycles, were performed by the DIL805 A/D quenching dilatometer from TA Instruments (Zaventem, Belgium), which included heating the samples in a low vacuum of 10^−4^ mbar to 900 °C at a heating rate of 10 °C/min and isothermal holding for 30 min on prepared cylindrical samples that were 10 mm in length and 4 mm in diameter. A calibrated S-type thermocouple was used for temperature measurement. Changes in length (∆*L*) and temperature (*T*) were registered during heating for every heating rate. The Savitzky–Golay method was applied as a signal smoothing processing.

Aging treatment of fabricated components was made in a high-temperature HT-2100 G-Vac Graphite-Special vacuum furnace from Linn High Therm GmbH (Eschenfelden, Germany). Samples were heated to a 480 °C at a heating rate of 10 °C/min with an isothermal holding in argon protective gas atmosphere for 5 h, then cooled in the furnace to ambient temperature.

X-ray diffraction (XRD) patterns were collected using an X-Pert PRO instrument (Eindhoven, The Netherlands). For X-ray diffraction analysis, with Cu target and a scan rate of 0.01 step/s, a scan range of 2θ of 30 to 110° were used. The influence of aging heat treatment on the SLM specimens was estimated to begin in specimens both in the as-built condition and following a standard isothermal aging treatment, performed at 480 °C for 5 h, followed by air quenching.

The hardness of the SLM components was measured by a Rockwell hardness tester (Zwick, Ulm, Germany), according to the 150 kg loaded Rockwell C scale (HRC), and estimated using an average value from 10 measured points.

## 3. Results and Discussion

Measurements from the technological properties of powder 18Ni-M300 steel showed that the flowability, according to the ISO 4497: 2020-04 standard [24], was 25 s and the bulk density, according to the ISO 3923-1:2018-09 standard [25], was found at 3.89 g/cm^3^, which tapped density according to the ISO 3953:2011standard [26] (4.85 g/cm^3^).

**Figure 1:**
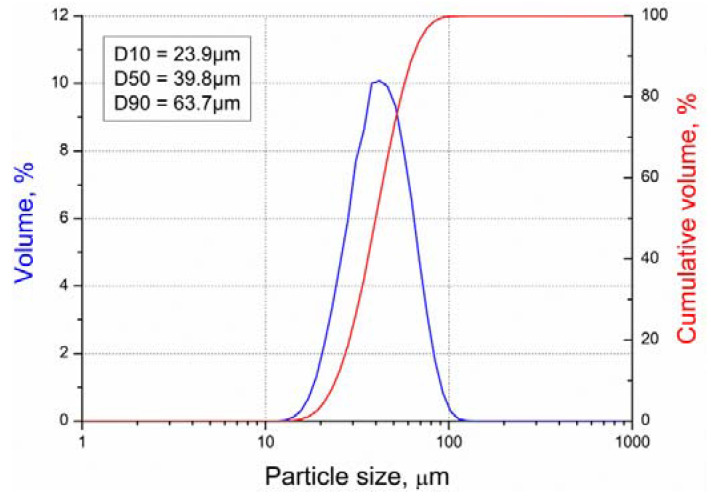
Particle size distributions of 18Ni-M300 powder.

The shape of particle size distribution was relatively narrow and nearly symmetric. The median diameter D50 was 39.8 µm, and D10–D90 diameter range was 23.9–63.7 µm (Figure 1).

**Figure 2:**
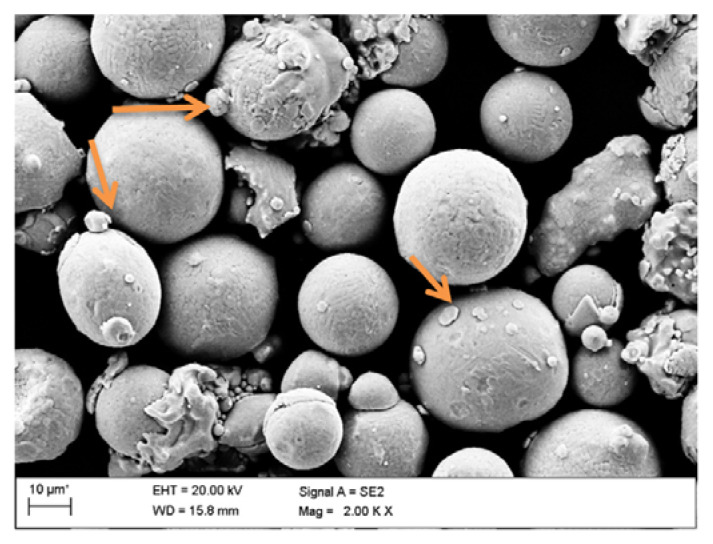
The microstructure of 18Ni-300 maraging steel powder.

Figure 2 presents the morphology of the delivered powder. The evaluation of the morphology of the powder revealed that not all particles were spherical and some of the powder grains included satellites marked with orange arrows.

**Figure 3:**
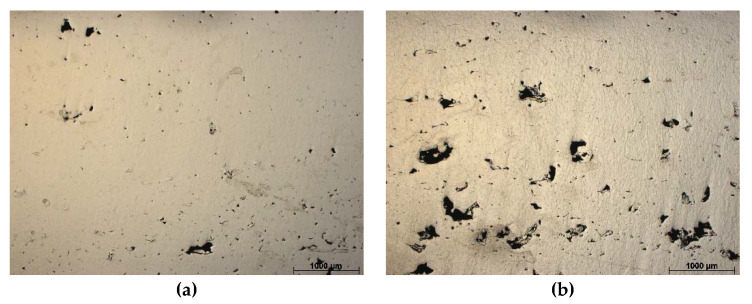

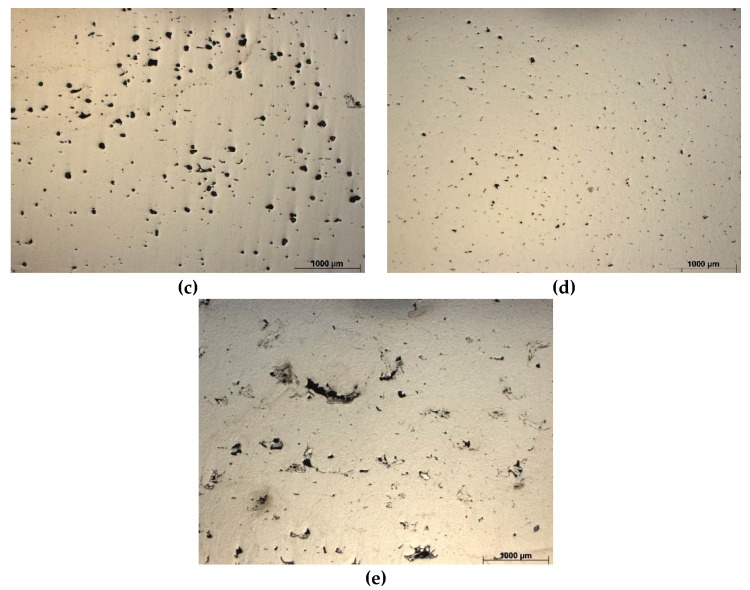
Light optical microscopy (LOM) images show defects in the horizontal cross-sections on analyzed material; (**a**–**e**) corresponds to sample numbers presented in Table 2.

**Figure 4:**
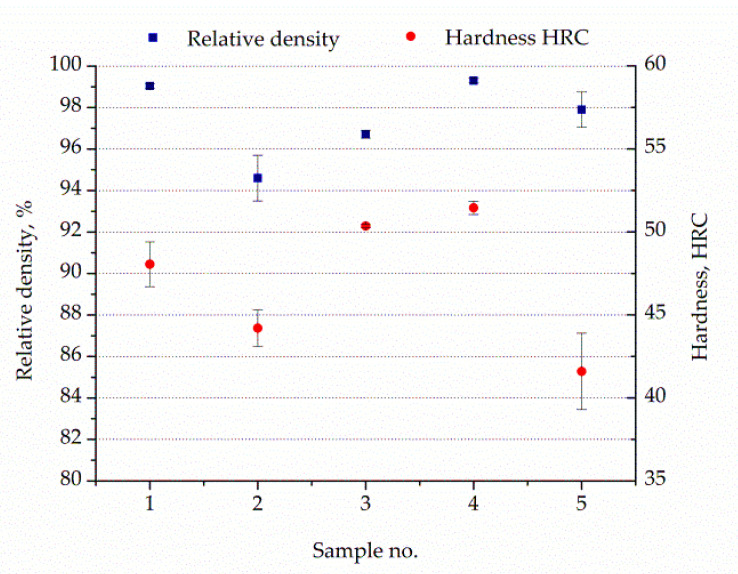
Relative density and hardness of the analyzed components after aging treatment.

Figure 3 illustrates the light optical microscopy (LOM) microstructures on the horizontal cross sections of samples after aging treatment, showing defects in appearance for keyholes, caves (Figure 3a,b,e), and gas pores (Figure 3c,d). The relative density values under different manufacturing conditions are presented in Figure 4. It can be noted that numerous defects can be found in the specimen built at a higher layer thickness, i.e., 50 µm.

At a constant energy density of approximately 160 J/mm^3^, numerous pores were visible. The low layer thickness, i.e., 30 µm, developed an extended melt pool and escaped from captured gases during the cooling sequence. These events in most round pores are presented in Figure 3c,d. Samples had relative density values of 96.7% and 99.3%, respectively. Components presented in Figure 3a,b,e exhibited extended and variable shaped pores caused by short laser exposure time, which affected the powder. It did not melt and presented holes in irregular shapes caused by particles removed the during preparing process. Samples were characterized by the relative density of 94.6–99.0%. The highest relative density of 99.3% was noted for components manufactured at 30 µm of layer thickness, 340 mm/s laser speed, and 120 µm of hatch distance, which was closer to the fully-dense bulk Ni-18 (M300). It must be noted that components in as-built condition, without aging treatment, were characterized by average hardness (approximately 40 HRC). Further, analyzed steel belongs to a special class of high-strength steel. It is different from conventional steel, in that it can be hardened by precipitation through a metallurgical reaction that does not include carbon.

Figure 4 presents changes in Rockwell hardness and relative density as a function of process conditions. It is clearly visible that porosity reflects on the hardness of analyzed components. Related to traditional maraging steel components with a hardness value of 28 HRC, all as-built components increased in hardness (41–51 HRC). The samples in as-built condition without aging treatment were characterized to have a hardness of 40 HRC, which is in agreement with the data provided by the producer. The highest hardness values for components after aging treatment were attributed to higher relative density achieved during fast cooling in the SLM technique.

Moreover, it can be observed from Figure 4 that Sample 1 had a higher relative density than Sample 3, but Sample 1 showed lower hardness than Sample 3. The occurred phenomenon in Sample 1 can be explained by occurrence defects having the shape of keyhole pores and caves caused by unmelted metal powder. Defects presented in Sample 3 had the form of gas porosity derived from metallurgical reactions (i.e., release of gasses). Differences in defect type can be related to layer thickness and hatch distance, and was higher in Sample 1 than in 3. The measured hardness was near to or slightly below 54 HRC [27].

**Figure 5:**
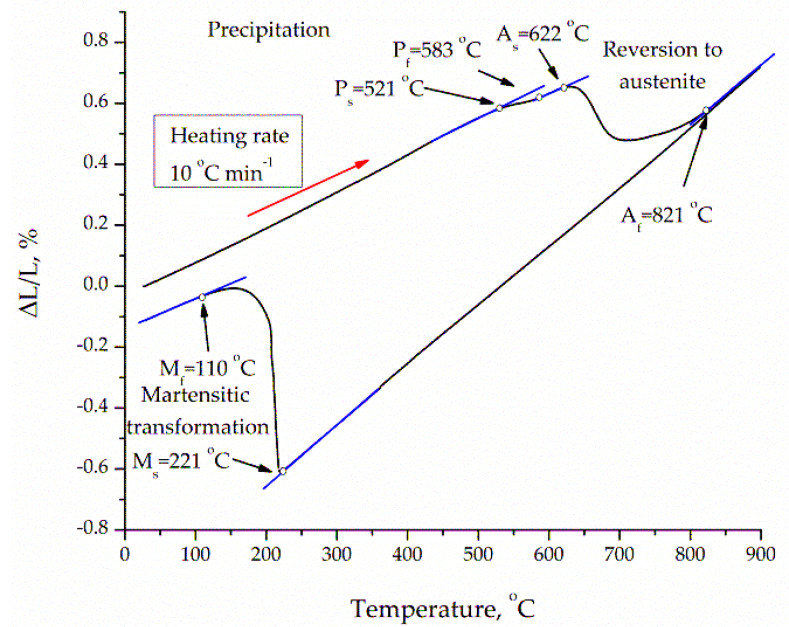
Variation in sample length at 10 °C/min for a SLM sample as a function of temperature [13].

Figure 5 presents a dilatometric graph of a component in an as-built state. The phase transition temperatures were determined based on inflections from tangents to the curve. Variations in sample length, ΔL, in relation to the initial length, l_o_, was a function of temperature. During the heating and cooling cycles, three-phase transitions were detected and recognized in 18Ni-300 steel. During the heating cycle, the first changes occurred at 521 °C on the angle slope of primary straight-line portion, which symbolized the start of precipitation (i.e., Ni_3_Ti and Ni_3_Mo phases followed by the Fe_2_Mo or Fe_7_Mo_6_ phases). The main strengthening precipitate process ended at 583 °C. The second deviation at 622 °C was caused by the austenite reversion *α*′ → *γ* transformation by shear and the dissolution of precipitates [15]. The austenite transformation finished at 821 °C. The third deviation occurred during the cooling cycle and corresponded to the martensitic transition, where M_s_ and M_f_ temperatures were 221 °C and 110 °C, respectively. Therefore, a single-phase microstructure characterized steel as martensite after cooling at room temperature [13].

The relatively soft martensite created upon cooling was hardened by the precipitation elements in a temperature range of 400–500 °C. Aging treatment below 450 °C afforded an organized and coherent precipitate, while fast and powerful aging treatment occurred from 450 and 600 °C. The isothermal aging treatment was done at a 480 °C temperature on the as-built components, to evaluate differences in the hardness.

**Figure 6:**
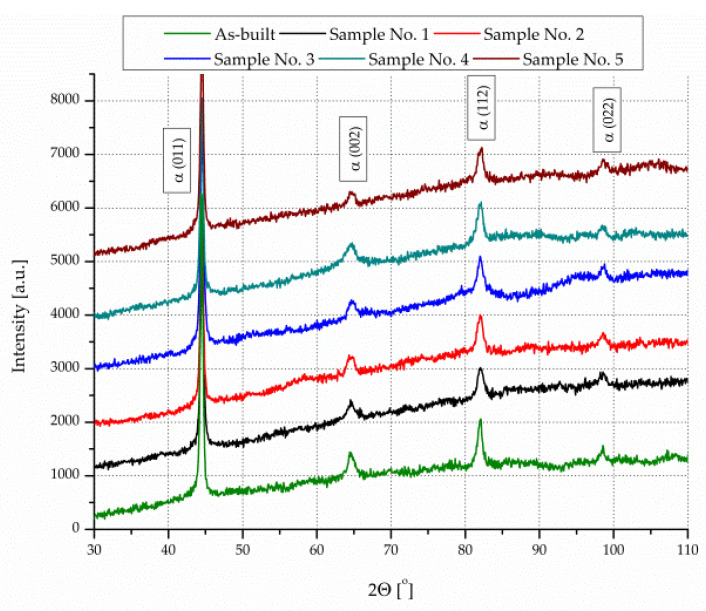
X-ray diffraction (XRD) patterns of 18Ni-300 steel components: as-built and aged heat treated.

Figure 6 presents XRD patterns for the as-built and aged heat treated components under various process conditions. The as-built component was featured in the martensite (*α*′) phase. When the aging treatment was at 480 °C for 5 h, the yet to be preserved *γ* phase existed in an aged specimen, i.e., α′ → *γ* phase transformation occurred as expected [28]. In addition, there was no clear change in the intensity of *α*′ phase orientation. Moreover, peaks from intermetallic phases, such as *η*-Ni_3_Ti, *γ*-Ni_3_Mo, *µ*-Fe_7_Mo_6_, and *σ*-FeMo, were not found in aged heat treated components. This probably correlated with the very small sizes of the intermetallic compounds.

**Figure 7:**
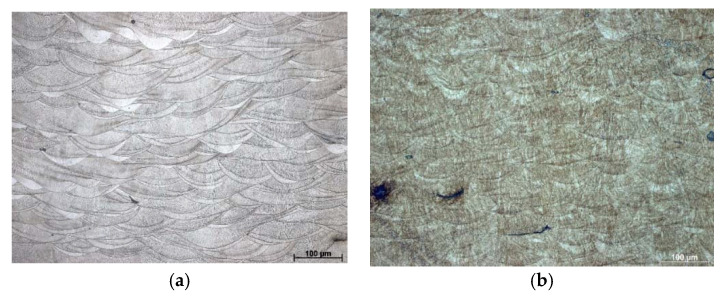
Representative sample: as-built (**a**) and after aging treatment (**b**).

Figure 7 presents the cross-sectional etched LOM microstructure on the as-built sample characterized by the highest relative porosity. The microstructure exhibited clear scan paths and the character of the melting process, as effected by a pulsating laser beam. In the cross-section, we recognized common semi-elliptical scan traces of crystallized melt pools with the long axis perpendicular to the laser beam, which overlapped between adjacent scan tracks. On the etched sections, the characteristics were visible and corresponded to the scanning parameters. Through the SLM process, the cooling speed varied inside the fluid pool; the highest cooling rate was located at the edge of the liquid pool where crystallization occurred faster than central areas. That effects of segregation in the fluid pool edges was evident following the etching process. Moreover, the laser scanning path changed by 67° following every layer, and it drove to uniquely oriented liquid pools. That exhibits on appearance and cross-sectioned liquid pool dimensions can be seen from the microstructure presented in Figure 7a. From the dimension (mainly the depth) of the layers, we assumed that every single laser scan included more than one single powder layer (the thickness of the deposited layer was 30 µm) and re-melting of the consolidated material happens. Through the SLM process, the thermal gradient was highest at the front boundaries of the laser beam, and the scan path growth was proportionate to a thermal grade. Hence, scan paths were frozen faster at the front border of the laser beam following their semi-elliptical contour. The shaded areas within scan paths exhibited heterogeneous dispersions in alloying and as-built components. The grains diffused off the grain edges due to a larger thermal gradient.

**Figure 8:**
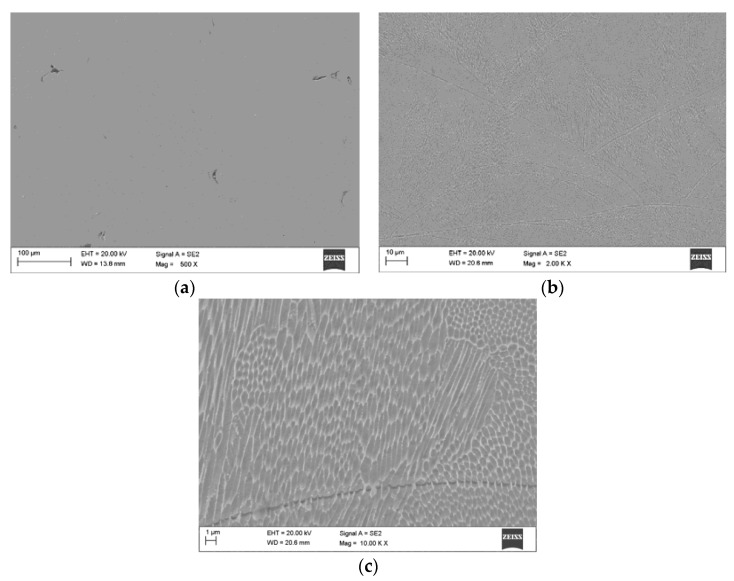
Microstructure of the as-built SLM Ni-18 (M300) steel during optimum process conditions (sample No. 4): (**a**) cross-sectioned scanning electron microscope (SEM) image with visible porosity, (**b**) SEM image after etching, and (**c**) the cellular structure of analyzed steel at high-magnification.

The microstructures of a cross-sectioned as-built SLM 18-Ni maraging steel (obtained during SEM analysis) are shown in Figure 8. It can be observed from Figure 8a that the SLM maraging steel showed a highly dense composition including few micro-pores. Holes and caves are typically generated by un-melted particles and capture of vapors in liquid pools through crystallization [28]. Figure 8b represents the etched cross-section and Figure 8c reveals the high-magnification of the grain microstructure for as-built 18Ni-300 steel. The structure of the as-fabricated M300 steel was represented in the shape of an excellent cellular formation. The aforementioned fine cellular formation is a typical structure of SLM steels, and it can affect the strength and hardness development of SLM steels as related to conventionally fabricated steels. The development of this type of structure is appropriate for high cooling rates in molten pools, which blocks the development of secondary dendrite arms through crystallization sequences. Moreover, high cooling speeds during crystallization were followed the development of α’ compound, and prevented the precipitation of intermetallic phases, rather than the alloying components, i.e., Ti, Co, Ni, and Mo, left in a supersaturated solid solution.

## 4. Conclusions

In the present study, we examined the influence of processing SLM parameters with constant energy density. Further, we analyzed aging heat treatment on the structure, relative density, hardness, and phase composition of SLM maraging 18Ni-300 steel. The main conclusions are as follows:

- A notable variation was determined in relative density as a result of the high intensity of pores and voids between scanning lines. The highest relative density, approximately 99.3%, was found for a manufactured component with 30 µm layer thickness, 340 mm/s laser speed, and 120 µm of hatch distance.
- The heating and cooling cycles analysis revealed three-phase transitions in analyzed maraging steel. The first deflection symbolized the beginning of precipitation, namely the Ni_3_Ti and Ni_3_Mo phases followed by the Fe_2_Mo or Fe_7_Mo_6_ phases. The second changes were caused via an austenite reversion *α*′ → *γ* transition. The third variations occurred during the cooling cycle and corresponded to the martensitic transition.
- The structural and X-ray diffraction analysis showed that the chosen temperature and aging time did not cause partial reversion of martensite into austenite.
- Applying the SLM process almost fully manufactured dens 18Ni-300 steel. The as-built SLM maraging steel had a well-made cellular structure.
- The strength of SLM maraging steel was improved through aging treatment. The high hardness of the SLM maraging steel was guaranteed by optimal aging condition (480 °C for 5 h in this work).

## Figures and Tables

**Table 1:** Comparison of the chemical composition of declared, real, and as-fabricated 18Ni-300.

Element, wt.%	Ni	Mo	Co	Ti	Cr	C	Si	Mn
Declared	17–19	4.5–5.2	8.5–10	0.6–1.2	<0.25	<0.03	<0.1	<0.15
Powder	17.8	4.5	8.6	1	0.11	<0.01	0.032	0.002
As-Fabricated	17.7	4.6	8.5	1	0.1	<0.01	0.033	<0.002

**Table 2:** Selective laser melting (SLM) process conditions.

Sample No.	Laser Speed, mm/s	Layer Thickness, µm	Hatch Distance, µm
1	200	50	120
2	270	50	90
3	480	30	90
4	340	30	120
5	300	50	80
